# Influence of visually perceived shape and brightness on perceived size, expected weight, and perceived weight of 3D objects

**DOI:** 10.1371/journal.pone.0220149

**Published:** 2019-08-08

**Authors:** Michele Vicovaro, Katia Ruta, Giulio Vidotto

**Affiliations:** Department of General Psychology, University of Padova, Padova, Italy; University of Exeter, UNITED KINGDOM

## Abstract

In the size–weight illusion, when two objects of identical weight but different volume are lifted, the smaller object is typically perceived to weigh more than the larger object. A well-known explanation for this and other weight illusions is provided by the hypothesis that perceived weight results from the contrast between actual and expected weight. More recently, it has been suggested that an object’s size may exert a direct and automatic effect on its perceived weight, independently of expected weight. Here we test these two hypotheses by exploring two illusions that have been known for a long time but have remained relatively underexplored, namely the shape–weight and brightness–weight illusions. Specifically, we measured the influence of visually perceived shape and brightness on the perceived size, the expected weight, and the perceived weight of 3D plastic objects. A numerical rating task was used in Experiment 1, and a paired comparison task was used in Experiment 2. The results showed that spheres were perceived to be heavier than tetrahedrons and cubes, and cubes were perceived to be heavier than tetrahedrons. We did not find any consistent relationship between brightness and perceived weight. A systematic comparison between perceived size, expected weight, and perceived weight showed that the visual shape–weight and brightness–weight illusions are partially inconsistent with the hypothesis that perceived weight results from the contrast between actual and expected weight and with the hypothesis that perceived weight results from the contrast between actual weight and perceived size. The results appear to suggest that there may be a dissociation between the processing of variables that contribute to the conscious experience of size, such as brightness and vertical height, and the processing of variables that contribute to perceived weight, such as surface area.

## Introduction

The idea that the subjective impression of weight is simply a function of physical weight has intuitive appeal, as it would mean that our sensory system works similarly to a weight scale. However, research has shown that the perceived weight of objects is actually affected by a variety of factors, such as the resistance of objects to rotation in space [1] and complex visual-motor relationships between motor commands and feedback signals [2,3,4]. Furthermore, research in the field of weight illusions has shown that perceived weight depends on various non-weight properties. For instance, when two objects of the same physical weight but different sizes are lifted, the smaller object usually feels heavier than the larger object, a phenomenon known as the size–weight illusion [5,6]. Another well-known illusion is the material–weight illusion, which refers to the phenomenon whereby, when two objects of the same physical weight but different surface materials are lifted, the object with the less dense surface material (e.g., polystyrene) usually feels heavier than the object with the denser surface material (e.g., iron [7,8,9]).

A unified explanation of weight illusions is provided by the so-called *expectation model* of weight perception [10,11], according to which perceived weight results from the contrast between sensory information about the actual weight of an object and cognitive information about its expected weight. Because of that contrast, whenever two objects of the same physical weight are expected to differ in weight, the object that is expected to be heavier is perceived to be lighter than the other object. Support for the expectation model has been provided by studies showing that weight expectations generated prior to the lifting action can directly cause the size–weight illusion [12] (cf. [13]) and the material–weight illusion [7] and by studies showing that the size–weight illusion can be inverted after extensive experience with objects that reverse the natural size-weight correlation [10,14,15]. Interestingly, Ellis and Lederman [16] showed that golfers had different expectations about the weights of golf balls with respect to non-golfers, and as a result, golfers and non-golfers had different perceptions of the weight of golf balls. In sum, there is converging evidence that expectations exert a top-down influence on weight perception (see [17]).

The size–weight illusion may result not only from top-down but also from bottom-up perceptual processes (see [1,17,18,19,20]). For instance, Ellis and Lederman [21] showed that the size–weight illusion is larger in magnitude when participants are asked to lift the stimuli by grasping them (i.e., the *haptic* size–weight illusion), as compared to when the stimuli are lifted by means of a string or a pulley (i.e., the *visual* size–weight illusion). Since somatosensory information about the rotational inertia [1] and density [20] of the stimuli is available when objects are grasped and lifted but not when objects are lifted by means of a string or a pulley, the fact that the haptic size–weight illusion is comparatively stronger than the visual size–weight illusion suggests that participants’ sensitivity to rotational inertia or density may contribute to the illusion. Despite this, the existence of the visual size–weight illusion indicates that the illusion can be generated by expectations alone [12] (cf. [13]).

In light of the results of previous studies, the idea that expectations can affect the perceived weight of objects can hardly be questioned. However, a challenge for the expectation model is provided in studies showing that, in some cases, perceived weight does not appear to be the result of the simple contrast between actual and expected weight [22,23,24,25]. For instance, Vicovaro and Burigana [25] presented the participants with pairs of cubic boxes varying in surface material and volume. In the expected weight condition, participants had to judge which stimulus appeared to be heavier, or if the two stimuli appeared to have the same weight (touching the stimuli was not allowed). In the perceived weight condition, the participants had to lift the stimuli using a string attached to the top surface in order to compare their perceived weight. The results showed that, inconsistently with the predictions from the expectation model, large stimuli made of a low-density surface material (i.e., polystyrene) were both expected and perceived to be lighter than small stimuli made of a relatively dense surface material (i.e., wood). In a similar vein, Dijker [23] found that large toy dolls that appeared to be weak in physical strength (e.g., Barbie) were both expected and perceived to be lighter than small toy dolls associated with apparently high physical strength (e.g., Action Man). A common feature of these two studies is that some of the large stimuli were expected and perceived to be lighter than some of the small stimuli (see also [22,24]). Consistent with this finding, Saccone and Choiunard [19] recently argued that, with respect to other non-weight properties, size has a unique ability to influence weight perception, and suggested that this might be due to the fact that size is processed rapidly before conscious awareness by the magnocellular pathway. Therefore, size would strongly and automatically affect perceived weight even when it exerts a relatively small influence on expected weight.

A contribution to the current debate on the relationship between perceived weight, expected weight, and perceived size [12,17,18,19,22,23,25] may come from the study of two illusions that have been known for a long time but that have remained relatively underexplored, namely the shape–weight illusion [26,27,28] and the brightness–weight illusion [29,30]. The main aim of the current study is to test the extent to which the two illusions can be explained in terms of the contrast between actual and expected weight, and/or in terms of the contrast between actual weight and perceived size. In other words, the shape–weight illusion and the brightness–weight illusion are here conceived as a test bench for two models of weight perception. A detailed review of the two illusions is provided in Sections 1.1 and 1.2 below.

### The shape–weight illusion

The first systematic study on the influence of the shape of objects on their perceived weight was conducted by Dresslar [26], who showed that shape had a systematic influence on perceived weight, and speculated that this was due to a size–weight illusion because the objects that were perceived to weigh more (e.g., the circle) were probably perceived to be smaller than the objects that appeared to weigh less (e.g., the triangle). Although Dresslar [26] did not measure the perceived size of the stimuli, if his hypothesis is correct, then the shape–weight illusion would simply be a by-product of the size–weight illusion, namely a size–weight illusion wherein differences in perceived size are due to differences in shape.

In a more recent study, Kahrimanovic, Bergmann Tiest, and Kappers [27] used 13 triplets of stimuli, each including three brass solids of identical physical mass and volume: a cube, a sphere, and a tetrahedron. The stimuli in each triplet differed from the stimuli in the other triplets both in physical mass and physical size. Blindfolded participants were asked to weigh one stimulus at a time by enclosing it with their hand and were allowed to perform up and down movement with the hand to perceive the weight. Results showed that the perceived weight of tetrahedrons was underestimated compared to that of cubes, whereas no difference in perceived weight emerged between tetrahedrons and spheres or between spheres and cubes. These results were replicated in Kahrimanovic, Bergmann Tiest, and Kappers’s [28] Experiment 1. In Experiment 2 of the latter study, in which the method of magnitude estimation was used instead of the method of paired comparisons, the results showed that tetrahedrons were perceived to be lighter than cubes and spheres. Again, no statistically significant difference emerged between cubes and spheres.

Using the same stimuli that they had used in their experiments on weight perception, Kahrimanovic, Bergmann Tiest, and Kappers [31] also measured the influence of shape on the haptically perceived size of objects. A direct comparison between the influence of shape on haptically perceived size and the influence of shape on haptically perceived weight showed that differences in perceived weight between tetrahedrons and cubes were actually larger than could be predicted from differences in perceived size [27,28]. Symmetrically, differences in perceived weight between tetrahedrons and spheres and between cubes and spheres were smaller than could be predicted from differences in perceived size. These results provide support for the hypothesis that the influence of shape on perceived weight is at least partially independent of the influence of shape on perceived size, which is inconsistent with Dresslar’s [26] hypothesis that the shape–weight illusion can be reduced to the size–weight illusion [27,28].

Because the expected weight of the stimuli was not measured in previous studies on the shape–weight illusion, it remains unclear whether the effect of shape on perceived weight could be explained in terms of the contrast between actual and expected weight [17,10,11]. The contribution of the current study to a deeper understanding of the shape–weight illusion can be summarized by two main points: 1) Whereas the studies conducted by Kahrimanovic, Bergmann Tiest, and Kappers [27,28] provide support to the existence of a *haptic* shape–weight illusion (i.e., participants enclosed the stimuli in the hand and could not see them), in the present study we test the existence of a *visual* shape–weight illusion, in that participants could see the stimuli but they could not touch them during the lifting action (i.e., participants were required to lift the stimuli by means of a string attached on the top). 2) By comparing the influence of shape on the perceived size, the expected weight, and the perceived weight of the stimuli, we could test whether the shape–weight illusion can be explained in terms of the contrast between actual and expected weight and/or in terms of the contrast between actual weight and perceived size, in line with the idea that perceived size may directly affect perceived weight independently of expected weight [19].

### The brightness–weight illusion

In his pioneering study on the influence of the color of objects on their perceived weight, De Camp [29] observed a weak positive relationship between the brightness of the stimuli and their perceived weight. For instance, a white cube tended to be judged as heavier than a black cube, and an orange cube tended to be judged as heavier than a blue cube. However, De Camp [29] himself found a somewhat opposite pattern of results in an experiment similar to the one just described in which participants were presented with three cubes varying in achromatic surface colors (i.e., white, gray, and black). Based on these conflicting results, De Camp [29] concluded that the influence of brightness on perceived weight was weak and inconsistent across the sets of stimuli, the participants, and the response methods (see also [32]). In a more recent study, Walker, Fracis, and Walker [30] presented participants with a set of eight cue snooker balls of equal size and mass, differing from each other in hue and brightness. The results showed a positive relationship between brightness and perceived weight: for instance, the white ball had a probability of about 0.8 to be judged as heavier than the black ball.

Whereas evidence of a relationship between brightness and *perceived* weight are somewhat mixed, there appears to exist converging evidence supporting a negative relationship between brightness and *expected* weight. For instance, in De Camp’s [29] study, a clear negative relationship emerged between brightness and visually estimated weight, as the light-colored objects were expected to be light in weight and the dark-colored objects were expected to be heavy in weight (see also [30, 33, 34]). Recently, Walker, Scallon, and Francis [35] provided support for the hypothesis that the correspondence between brightness and weight is bidirectional; that is, not only brightness affects expected weight but perceived weight affects expected brightness. The authors asked participants to rate the expected brightness of unseen lifted objects, and the results showed that heavy-feeling objects were expected to be darker than light-feeling objects. Overall, the results of these studies suggest that the brightness–weight illusion may stem from the contrast between actual and expected weight (i.e., dark objects are expected to weigh more, and therefore they are perceived to weigh less than bright objects).

Interestingly, the brightness–weight illusion appears to be at least partially inconsistent with the hypothesis that perceived size can directly and automatically affect perceived weight independently of expected weight [19]. Indeed, previous studies show that the apparent size of objects tends to increase with their brightness (i.e., light objects are perceived to be larger than dark objects; see [36,37,38]), therefore if perceived size exerted a direct influence on perceived weight, then perceived weight should decrease–rather than increase–with brightness. In other words, the brightness–weight illusion appears to be more consistent with the predictions of the expectation model than with the hypothesis of a direct and automatic influence of perceived size on perceived weight. The contribution of the current study to a deeper understanding of the brightness–weight illusion can be summarized in two points: 1) To the best of our knowledge, only one study has so far provided convincing support for the existence of a brightness–weight illusion [30]. Here we test the replicability and generalizability of the brightness–weight illusion using a set of stimuli, a weighing mode, and a psychophysical method different from those originally employed by Walker, Francis, and Walker [30]. 2) By comparing the influence of brightness on the perceived size, the expected weight, and the perceived weight of the stimuli, we could test the extent to which the brightness–weight illusion can be explained in terms of the contrast between actual and expected weight and/or in terms of the contrast between actual weight and perceived size.

## Experiment 1

### Participants

Thirty-four right-handed participants (15 females, 19 males) took part in the experiment in exchange for course credit. They were undergraduate students at the University of Padua ranging in age from 21 to 43 years (*M* = 24.94, *SD* = 3.87). All had normal or corrected-to-normal vision and normal function of their upper limbs. They were naive as to the purposes of the experiment. All the participants took part in the *expected weight* and the *perceived weight* sessions (see below), and a subset of 20 participants (11 females, 9 males; mean age = 24.4, *SD* = 2.56) also took part in the *perceived size* session.

### Stimuli and design

**Fig 1:**
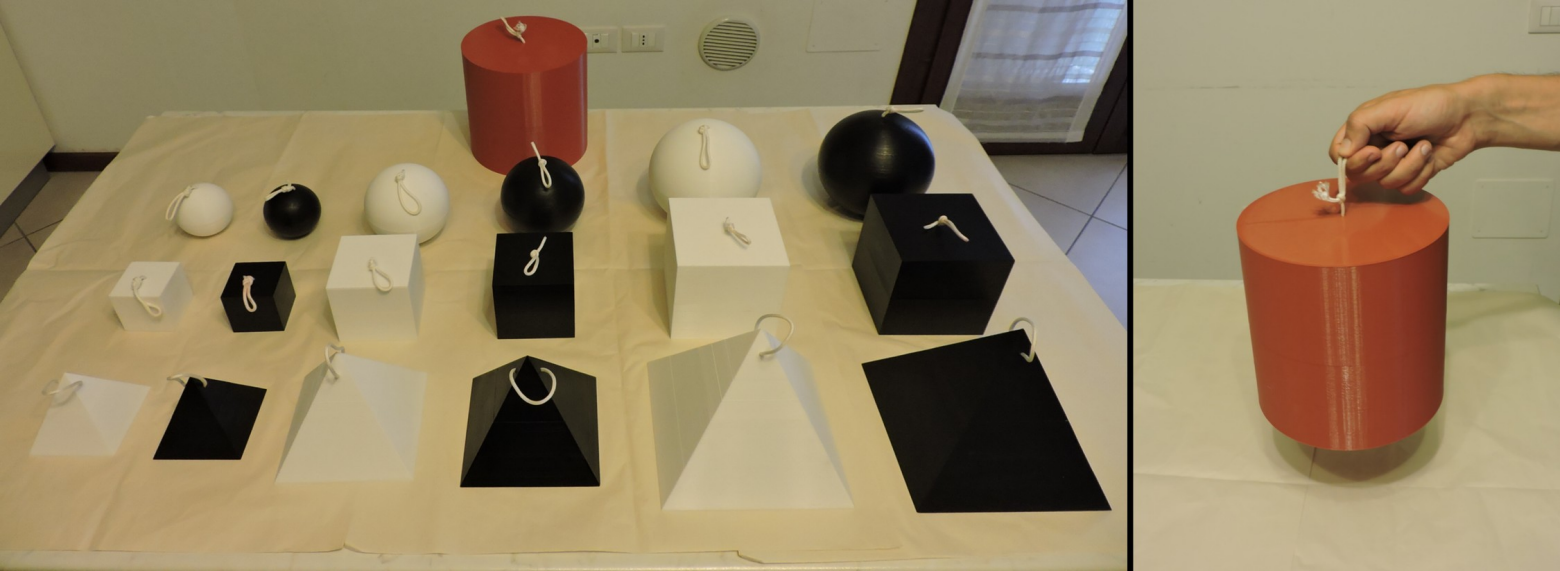
Left panel: a picture of the stimuli used in Experiment 1. Right panel: a depiction of the lifting action.

The stimuli were 18 plastic 3-D objects each weighing 220 g that were built according to a 3 (Size) × 3 (Shape) × 2 (Brightness) factorial design. All the stimuli were built using a Zmorph3D printer and could be opened and filled with a variable amount of cotton wool and plasticine, which allowed us to regulate their mass. When closed, all the stimuli had a uniform appearance (see Fig 1). The volume of the stimuli could be 216, 729, or 1728 cc. The shapes could be tetrahedron, cube, or sphere (detailed measures of the stimuli are reported in Table 1). The color of the stimuli could be white or black (in the room where the experiment took place, the average luminance measured using a Minolta LS-100 photometer was 3.82 cd/m^2^ for the black stimuli and 49.85 cd/m^2^ for the white stimuli). An orange 4580 cc, 440 g cylinder (height = diameter = 18 cm, luminance = 20.72 cd/m^2^) served as the standard stimulus (see Fig 1). A thin string (diameter = 0.3 cm) was looped through holes in the top of the experimental stimuli and of the standard stimulus (see Fig 1). Due to the technical features of the stimuli, the method of attaching the strings was different across the three shapes. For tetrahedrons, the string formed a loop passing through two holes close to the apex; for cubes, spheres, and the cylinder, the string was glued to the inside of a small hole in the top surface of the object, and the loop was formed making a knot to the external part of the string (see Fig 1). The overall height of the loop from the surface of the stimulus was about 5 cm, and all the stimuli could be lifted easily with the thumb and index fingers (see Fig 1, left panel). The lifting action did not vary with the method of attaching strings to the stimuli, therefore the latter variable should have a negligible influence on the forces with which the stimuli were lifted.

**Table 1:** Detailed measures of the stimuli.

Volume	Tetrahedron (base side)	Tetrahedron (height)	Cube (side)	Sphere (diameter)
216 cc	8.65 cm	8.65 cm	6 cm	7.45 cm
729 cc	13 cm	13 cm	9 cm	11.2 cm
1728 cc	17.3 cm	17.3 cm	12 cm	14.9 cm

There were three experimental sessions, which we call the *perceived size* session, the *expected weight* session, and the *perceived weight* session. The perceived size and expected weight sessions lasted about 20–25 minutes, whereas the perceived weight session lasted about 25–30 minutes. The sessions took place at least two days apart. Half of the 20 participants who took part in all three sessions first completed the perceived size session, then the expected weight session, and then the perceived weight session; the order of the first two sessions was inverted for the other half of these 20 participants. All the participants who took part in only two experimental session first completed the expected weight session and then the perceived weight session.

In each session, the participants were presented with four repetitions of the 18 experimental stimuli and of the standard stimulus. The responses in the first repetition were considered practice and therefore were not analyzed. Each repetition started with the presentation of the standard stimulus followed by the randomized presentation of the 18 experimental stimuli. As detailed below, in each of the three sessions, a numerical rating response method with fixed anchors was used. Specifically, participants were asked to rate the perceived size, the expected weight, or the perceived weight of each experimental stimulus relative to the extremes of the rating scale, which were 0 (i.e., the absence of size or weight) and 100 (i.e., the size or the weight of the standard). Numerical rating tasks with fixed anchors provided measures on an interval scale of the response variable [39,40], and had been previously employed to measure the magnitude of the size–weight illusion [13,41].

### Procedure

In each experimental session, the participants were seated at a square table (height 85 cm) with their elbows leaning on it. Before starting the first experimental session, they read and signed an informed consent form approved by the local ethics committee (Department of General Psychology, University of Padua). All procedures performed were in accordance with the ethical standards of the 1964 Helsinki declaration and its later amendments. The experimenter sat in front of the participant. A cardboard screen was used to hide from participant the positioning and removal of the stimuli, which were manually performed by the experimenter. The stimuli were placed on a thin layer of soft rubber to muffle the noise of the contact of the stimuli with the hard surface of the table. To warrant the equilibrium of the sphere, a small hollow was created in the rubber layer exactly in where the stimuli were placed by the experimenter. Once the stimulus was set into place by the experimenter, the occluding screen was removed and the participant was allowed to evaluate the perceived size, the expected weight, or the perceived weight of the stimulus, as detailed below. The stimuli were placed at a distance of about 30 cm from the side of the table where the participant was seated. After the participant had responded, the experimenter reset the occluding screen in its position, removed the previous stimulus from the table, and placed a new stimulus on the table.

At the beginning of the perceived size session, written instructions informed the participants that they would be presented with an orange cylinder, the size of which was 100. They were then informed that, after the presentation of the cylinder, stimuli varying in volume, shape, and color would have been presented, and that their task was to evaluate the size of each stimulus using a number between 0 and 100, where 0 would be the size of an invisible object, and 100 was the size of the cylinder. The instructions further specified that neither the cylinder nor the other stimuli could be touched at any moment during the experiment. In the expected weight session, the instructions were the same as those in the perceived size session, except that participants were informed that their task was to visually estimate the weight of each stimulus using a number between 0 and 100, where 0 was the absence of weight and 100 was the apparent weight of the cylinder. In the perceived weight session, participants were instructed to lift the stimuli (including the standard) by grasping the string on their tops using the thumb and index fingers of their right hands. The participants were further instructed to keep their elbows on the table during the lifting action, to avoid oscillations of the stimuli as much as possible, and to respond only after the lifting action had been completed. This procedure should prevent or minimize access to information about the rotational inertia of the stimuli. Participants were then informed that their task was to evaluate the weight of each object using a number between 0 and 100, where 0 was the absence of weight and 100 was the weight of the cylinder.

### Results and discussion

**Fig 2:**
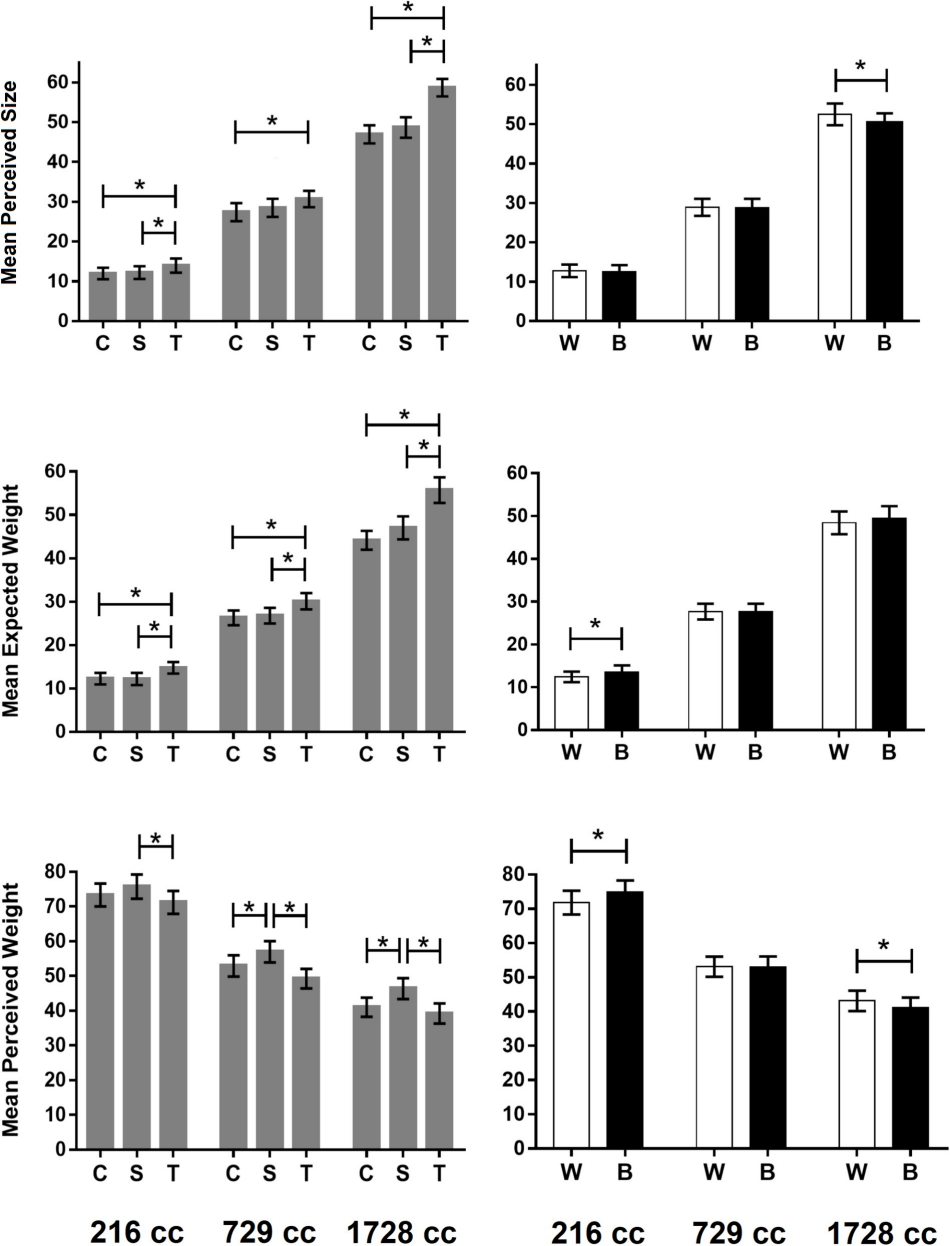
The results of Experiment 1. Top, middle, and bottom panels represent mean perceived size, mean expected weight, and mean perceived weight, respectively. In the left panels, the means of the response variable are represented as a function of the three levels of volume and as a function of the three shapes. The letters C, S, and T stand for cube, sphere, and tetrahedron, respectively. In the right panels, the means are represented as a function of the three levels of volume and as a function of the two levels of brightness. The letters W and B stand for white and black, respectively. The error bars represent the standard error of the mean. The * symbol indicates a statistically significant comparison (*p* < .05; Bonferroni correction method was applied).

Fig 2 shows the mean perceived size (top panels), the mean expected weight (middle panels), and the mean perceived weight (bottom panels) of the stimuli, averaged across participants and repetitions as a function of volume and shape (left panels) and as a function of volume and brightness (right panels). As detailed below, perceived size, expected weight, and perceived weight were analyzed using separate ANOVAs.

#### Perceived size

We performed a three-way within-participants ANOVA in perceived size with the factors volume, shape, and brightness. There were main effects of all three factors (volume: *F*(2,38) = 416.43, *p* < .001, *η*_*G*_^2^ = 0.92; shape: *F*(2,38) = 37.99, *p* < .001, *η*_*G*_^2^ = 0.21; brightness: *F*(1,19) = 6.51, *p* = .019, *η*_*G*_^2^ = 0.007). The volume × shape and volume × brightness interactions were also statistically significant (*F*(4,76) = 14.76, *p* < .001, *η*_*G*_^2^ = 0.14; *F*(2,38) = 4.54, = .017, *η*_*G*_^2^ = 0.01 respectively). The shape × brightness interaction and the three-way interaction were not statistically significant (*F*(2,38) = 1.03, *p* > .10, *η*_*G*_^2^ = 0.001; *F*(4,76) = 0.67, *p* > .10, *η*_*G*_^2^ = 0.002 respectively).

Effects of volume. Post hoc paired comparisons tests with Bonferroni correction method showed that large stimuli were perceived to be significantly larger than medium and small stimuli (*t*(19) = 17.08, *p* < .001, *d*_*z*_ = 3.82; *t*(19) = 24.77, *p* < .001, *d*_*z*_ = 5.54 respectively), and medium stimuli were perceived to be significantly larger than small stimuli (*t*(19) = 14.39, *p* < .001, *d*_*z*_ = 3.22).

Effects of shape. We further explored the main effects of *shape* on perceived size using corrected post hoc paired comparisons tests. The results showed that tetrahedrons were perceived to be larger than spheres (*t*(19) = 6.26, *p* < .001, *d*_*z*_ = 1.4) and cubes (*t*(19) = 6.64, *p* < .001, *d*_*z*_ = 1.48) and that spheres tended to be perceived as slightly larger than cubes, although the difference was not statistically significant (*t*(19) = 2.49, *p* = .066, *d*_*z*_ = 0.56). Regarding the volume × shape interaction, the top left panel in Fig 2 clearly shows that the effect of shape on perceived size tended to increase with physical size. Moreover, corrected post hoc paired comparisons showed that tetrahedrons were perceived to be significantly larger than spheres for the small and the large set (small set: *t*(19) = 5.0, *p* < .001, *d*_*z*_ = 1.12; medium set: *t*(19) = 2.75, *p* > .10, *d*_*z*_ = 0.61; large set: *t*(19) = 5.38, *p* < .001, *d*_*z*_ = 1.20), that tetrahedrons were perceived to be significantly larger than cubes at all the levels of volume (small set: *t*(19) = 3.74, *p* = .012, *d*_*z*_ = 0.84; medium set: *t*(19) = 3.7, *p* = .014, *d*_*z*_ = 0.83; large set: *t*(19) = 5.89, *p* < .001, *d*_*z*_ = 1.32), and that no statistically significant differences emerged between spheres and cubes at any of the levels of volume (small set: *t*(19) = 0.73, *p* > .10, *d*_*z*_ = 0.16; medium set: *t*(19) = 2.0, *p* = > .10, *d*_*z*_ = 0.45; large set: *t*(19) = 1.43, *p* > .10, *d*_*z*_ = 0.32).

Effects of brightness. The ANOVA shows a main effect of the brightness factor. This is due to the fact that, on average, white stimuli were perceived to be larger than black stimuli. However, the significant volume × brightness interaction suggests that the effects of brightness on perceived size were modulated by physical volume. Corrected post-hoc paired comparisons showed that white stimuli were perceived to be significantly larger than black stimuli only for the large set (small set: *t*(19) = 0.81, *p* > .10, *d*_*z*_ = 0.18; medium set: *t*(19) = 0.28, *p* > .10, *d*_*z*_ = 0.06; large set: *t*(19) = 2.59, *p* = .049, *d*_*z*_ = 0.58). Overall, the results of the perceived size session confirm the existence of a positive relationship between brightness and perceived size, which had been previously reported for two-dimensional stimuli [36,37,38]. The results also suggest that the influence of brightness on perceived size tended to increase with volume.

#### Expected weight

We performed a three-way within-participants ANOVA in expected weight with the factors volume, shape, and brightness. There were main effects of volume and shape (*F*(2,66) = 267.09, *p* < .001, *η*_*G*_^2^ = 0.83; *F*(2,66) = 64.7, *p* < .001, *η*_*G*_^2^ = 0.13, respectively), but not of brightness (*F*(1,33) = 1.75, *p* > .10, *η*_*G*_^2^ = 0.004). The volume × shape and shape × brightness interactions were statistically significant (*F*(4,132) = 17.19, *p* < .001, *η*_*G*_^2^ = 0.06; *F*(2,66) = 4.06, *p* = .022, *η*_*G*_^2^ = 0.003, respectively). The volume × brightness interaction and the three-way interaction were not statistically significant (*F*(2,66) = 1.26, *p* > .10, *η*_*G*_^2^ = 0.002; *F*(4,132) = 0.92, *p* > .10, *η*_*G*_^2^ = 0.001, respectively).

Effects of volume. Post hoc paired comparisons tests with Bonferroni correction method showed that large stimuli were expected to be significantly heavier than medium and small stimuli (*t*(33) = 14.92, *p* < .001, *d*_*z*_ = 2.56; *t*(33) = 17.09, *p* < .001, *d*_*z*_ = 2.93 respectively), and medium stimuli were expected to be significantly heavier than small stimuli (*t*(33) = 15.65, *p* < .001, *d*_*z*_ = 2.68).

Effects of shape. We further explored the main effects of shape using corrected post hoc paired comparisons tests. The results showed that tetrahedrons were expected to be heavier than spheres (*t*(33) = 9.48, *p* < .001, *d*_*z*_ = 1.62) and cubes (*t*(33) = 8.65, *p* < .001, *d*_*z*_ = 1.48), and that spheres were generally expected to be slightly heavier than cubes, although the difference was not statistically significant (*t*(33) = 2.49, *p* = .053, *d*_*z*_ = 0.43). Regarding the volume × shape interaction, the middle left panel in Fig 2 clearly shows that the effect of shape on expected weight tended to increase with physical volume. Moreover, corrected post hoc paired comparisons showed that, at all levels of volume, tetrahedrons were expected to be significantly heavier than spheres (small set: *t*(33) = 4.63, *p* < .001, *d*_*z*_ = 0.79; medium set: *t*(33) = 5.12, *p* < .001, *d*_*z*_ = 0.88; large set: *t*(33) = 7.87, *p* < .001, *d*_*z*_ = 1.35), and cubes (small set: *t*(33) = 4.31, *p* = .00014, *d*_*z*_ = 0.74; medium set: *t*(33) = 5.65, *p* < .001, *d*_*z*_ = 0.97; large set: *t*(33) = 7.03, *p* < .001, *d*_*z*_ = 1.21). No statistically significant differences emerged between spheres and cubes at any of the levels of volume (small set: *t*(33) = -0.29, *p* > .10, *d*_*z*_ = -0.05; medium set: *t*(33) = 1.0, *p* > .10, *d*_*z*_ = 0.17; large set: *t*(33) = 2.40, *p* > .10, *d*_*z*_ = 0.41).

The comparison between the top and middle left panels in Fig 2 shows that shape had very similar effects on perceived size and expected weight, which may suggest that the influence of shape on expected weight was largely or totally mediated by perceived size. To further explore this hypothesis, we performed a hierarchical linear regression analysis on expected weight using mixed-effects models (R package *lme4* [42]). Specifically, we fit six nested linear mixed-effects models of increasing complexity to the data of the expected weight sessions, limited to the data of the 20 participants who also took part in the perceived size session. Each model included all the features of the models preceding it in the hierarchy of complexity plus some additional feature (see Table 2). For each model, Table 2 shows two indexes of goodness of fit, namely the Akaike information criterion (AIC) and the Bayesian information criterion (BIC), as well as the value of the chi-square statistic for the log-likelihood ratio test comparing that model with the model preceding it in the hierarchy of complexity (together with the associated *p*-value). If *p* < .05, then the more complex model (e.g., Model 3) fits the data significantly better than the simpler model (Model 2). The results show that the model including perceived size and shape as fixed effects (i.e., Model 4) did not fit the data significantly better than the model including perceived size alone as a fixed effect (i.e., Model 3). This suggests that when the effects of perceived size on expected weight were taken into account, shape did not affect expected weight directly. In other words, the influence of shape on expected weight was fully mediated by the influence of shape on perceived size.

**Table 2:** Results of the hierarchical regression analysis on expected weight (mixed models). Bold typeface indicates the model with the best fit to the data according to the log-likelihood ratio test.

N.	Random effect(s)	Fixed effect(s)	AIC	BIC	Chi-square	*p*-value
1	Intercept	-	9320.7	9335.7		
2	Intercept	Perc. size	7961.7	7981.7	χ^2^(1) = 1361	< .001
**3**	**Intercept,** **Perc. size (slope)**	**Perc. size**	**7633.9**	**7663.8**	**χ**^**2**^**(2) = 331.8**	**< .001**
4	Intercept, Perc. size (slope)	Perc. size, Shape	7632.0	7671.8	χ^2^(2) = 5.968	.051
5	Intercept, Perc. size (slope), Shape (slope)	Perc. size, Shape	7638.2	7708.0	χ^2^(6) = 5.759	> .10
6	Intercept, Perc. size (slope), Shape (slope)	Perc. size × Shape	7640.0	7719.8	χ^2^(2) = 2.178	> .10

Effects of brightness. Brightness did not appear to have a consistent effect on the expected weight of the stimuli (see the middle right panel in Fig 2). Besides the lack of main effect of the brightness factor, a Bayesian paired-sample *t*-test provided support for the null hypothesis of equivalence between black and white stimuli in expected weight, since the JZS BF_01_ = 2.45 ± 0%. Although the volume × brightness interaction was not statistically significant, for the sake of comparison with the results of the perceived size session, we further explored the interaction using paired comparison tests with Bonferroni correction. These comparisons showed that, for the small set, black stimuli were expected to be significantly heavier than white stimuli (*t*(33) = 2.62, *p* = .04, *d*_*z*_ = 0.45). No statistically significant difference emerged between black and white stimuli for the medium and large sets (*t*(33) = 0.09, *p* > .10, *d*_*z*_ = 0.02; *t*(33) = 1.04, *p* > .10, *d*_*z*_ = 0.18, respectively). Although the shape × brightness interaction was statistically significant, post-hoc paired comparisons tests with Bonferroni correction failed to reveal any statistically significant difference between black and white stimuli at any level of shape (tetrahedrons: *M*_*black*_ = 34.26, *M*_*white*_ = 32.78, *t*(33) = 2.10, *p* > .10, *d*_*z*_ = 0.36; spheres: *M*_*black*_ = 29.19, *M*_*white*_ = 28.12, *t*(33) = 1.47, *p* > .10, *d*_*z*_ = 0.25; cubes: *M*_*black*_ = 27.45, *M*_*white*_ = 27.65, *t*(33) = -0.33, *p* > .10, *d*_*z*_ = -0.06).

The lack of an overall effect of brightness on expected weight is inconsistent with the results of previous studies [29,30,33,34]. A plausible explanation for this finding is that, in the current study, the negative relationship between brightness and expected weight was overridden by the positive relationship between brightness and perceived size (i.e., white stimuli were perceived to be larger, so they were expected to be slightly heavier than black stimuli). Support for this hypothesis is provided by the fact that black stimuli were expected to be significantly heavier than white stimuli only for the small set (see the middle-right panel in Fig 2), namely the level of volume at which the influence of brightness on perceived size was at its minimum (see the top-right panel in Fig 2). We can speculate that in Experiment 1, the variations in perceived volume were made particularly salient by the manipulation of volume, which might explain why the effects of shape and brightness on expected weight were strongly mediated by perceived size. An indirect test of this hypothesis will be provided in Experiment 2.

#### Perceived weight

We performed a three-way within-participants ANOVA in perceived weight with factors volume, shape, and brightness. Main effects existed for the factors volume and shape (*F*(2,66) = 101.67, *p* < .001, *η*_*G*_^2^ = 0.65; *F*(2,66) = 19.6, *p* < .001, *η*_*G*_^2^ = 0.07, respectively) but not for brightness (*F*(1,33) = 0.5, *p* > .10, *η*_*G*_^2^ < 0.001). The volume × brightness and shape × brightness interactions were statistically significant (*F*(2,66) = 10.79, *p* < .001, *η*_*G*_^2^ = 0.012; *F*(2,66) = 8.54, *p* < .001, *η*_*G*_^2^ = 0.008, respectively). The volume × shape interaction was not statistically significant (*F*(4,132) = 1.69, *p* > .10, *η*_*G*_^2^ = 0.005), whereas the three-way interaction was statistically significant (*F*(4,132) = 5.81, *p* < .001, *η*_*G*_^2^ = 0.013).

Effects of volume. Consistent with the size–weight illusion, there was a strong negative relationship between volume and perceived weight. Corrected post hoc paired comparisons showed that small stimuli were perceived to be significantly heavier than medium and large stimuli (*t*(33) = 10.1, *p* < .001, *d*_*z*_ = 1.73; *t*(33) = 10.39, *p* < .001, *d*_*z*_ = 1.78 respectively), and medium stimuli were perceived to be significantly heavier than large stimuli (*t*(33) = 8.24, *p* < .001, *d*_*z*_ = 1.41).

Effects of shape. Corrected post hoc paired comparisons showed that spheres were perceived to be heavier than tetrahedrons (*t*(33) = 5.28, *p* < .001, *d*_*z*_ = 0.9) and cubes (*t*(33) = 3.6, *p* = .0031, *d*_*z*_ = 0.62) and that cubes were perceived to be heavier than tetrahedrons (*t*(33) = 3.39, *p* = .0054, *d*_*z*_ = 0.58). For each level of the volume factor, the results of the statistically significant paired comparison tests between shapes (with Bonferroni correction) are shown in the bottom-left panel in Fig 2. The observed shape–weight illusion was not fully consistent with the predictions from the expectation model. Indeed, if the shape–weight illusion resulted from the contrast between actual and expected weight, then because the spheres were generally expected to be slightly heavier than cubes, they should have been perceived to be slightly lighter–rather than heavier–than cubes. Moreover, because the differences between the three shapes in expected weight tended to increase with volume, the influence of shape on perceived weight should also have increased with volume. The lack of a statistically significant volume × shape interaction suggests that this was not the case. We fit four nested linear mixed-effects models of increasing complexity to the data from the perceived weight session for all 34 participants (see Table 3). Models with complexity higher than that of Model 4 failed to converge; thus, they were removed from the analysis. The results show that the influence of shape on perceived weight was at least partially independent of the influence of shape on expected weight, since shape had a statistically significant effect on perceived weight, even when the effects of expected weight on perceived weight were taken into account.

**Table 3:** Results of the hierarchical regression analysis on perceived weight (mixed models). Bold typeface indicates the model with the best fit to the data, according to the log-likelihood ratio test.

N.	Random effect(s)	Fixed effect(s)	AIC	BIC	Chi-square	*p*-value
1	Intercept	-	16185	16201		
2	Intercept	Exp. w.	15342	15364	χ^2^(1) = 845.18	< .001
3	Intercept, Exp. w. (slope)	Exp. w.	15104	15137	χ^2^(2) = 241.77	< .001
**4**	**Intercept,** **Exp. w. (slope)**	**Exp. w., Shape**	**15075**	**15120**	**χ**^**2**^**(2) = 32.52**	**< .001**

The results are also somewhat inconsistent with the hypothesis that perceived size would exert a direct and automatic influence on perceived weight [19]. Indeed, if perceived weight depended on the contrast between actual weight and perceived size, then because spheres were perceived to be slightly larger than cubes, spheres should have been perceived as being slightly lighter–rather than heavier–than cubes. Additionally, the influence of shape on perceived weight should have increased with volume, but this was not the case. We fit four nested linear mixed-effects models of increasing complexity to the data from the perceived weight session, limited to the data of the 20 participants who also took part in the perceived size session (see Table 4). Models with complexity higher than that of Model 4 failed to converge; thus, they were removed from the analysis. The results show that the influence of shape on perceived weight was at least partially independent of the influence of shape on perceived size, since shape had a statistically significant effect on perceived weight, even when the effects of perceived size on perceived weight were taken into account.

**Table 4:** Results of the hierarchical regression analysis on perceived weight (mixed models). Bold typeface indicates the model with the best fit to the data, according to the log-likelihood ratio test.

N.	Random effect(s)	Fixed effect(s)	AIC	BIC	Chi-square	*p*-value
1	Intercept	-	9367	9382		
2	Intercept	Perc. size	8836	8856	χ^2^(1) = 533.74	< .001
3	Intercept, Perc. size (slope)	Perc. size	8767	8797	χ^2^(2) = 72.64	< .001
**4**	**Intercept,** **Perc. size (slope)**	**Perc. size, Shape**	**8751**	**8792**	**χ**^**2**^**(2) = 19.33**	**< .001**

Effects of brightness. As for the effects of brightness on perceived weight, the main effect of the brightness factor was not statistically significant, and a Bayesian paired-sample *t*-test provided support for the null hypothesis of equivalence between black and white stimuli in perceived weight, since JZS BF_01_ = 4.35 ± 0%. However, the bottom-right panel in Fig 2 reveals a surprising pattern of results, which can explain the statistical significance of the volume × brightness interaction. Specifically, paired-comparison tests with Bonferroni correction showed that black stimuli were perceived to be significantly heavier than white stimuli for the small set (*t*(33) = 3.73, *p* = .0021, *d*_*z*_ = 0.64), whereas white stimuli were perceived to be significantly heavier than black stimuli for the large set (*t*(33) = 2.82, *p* = .024, *d*_*z*_ = 0.48). No statistically significant difference in perceived weight emerged between white and black stimuli for the medium set (*t*(33) = 0.1, *p p* > .10, *d*_*z*_ = 0.02). In other words, a regular brightness-weight illusion emerged for large stimuli, whereas an inverted illusion emerged for small stimuli. Importantly, both the regular and the inverted brightness-weight illusions appear to be inconsistent with the predictions from the expectation model. Specifically, the results of the expected weight session showed that, for the small set, black stimuli were expected to be heavier than white stimuli; therefore, a regular rather than an inverted brightness–weight illusion should have appeared. Moreover, for the large set, a regular brightness–weight illusion emerged, despite the lack of difference in expected weight between black and white stimuli. The observed influence of brightness on perceived weight was also inconsistent with the idea that perceived size exerted a direct influence on perceived weight. Indeed, the results of the perceived size session showed that, for the large set, white stimuli were perceived to be larger than black stimuli; hence, an inverted–rather than a regular–brightness–weight illusion should have appeared. Moreover, no influence of brightness on perceived weight should have emerged for the small set, since there was no difference in perceived size between black and white stimuli at that volume level.

#### Effects of brightness: Individual differences

**Fig 3:**
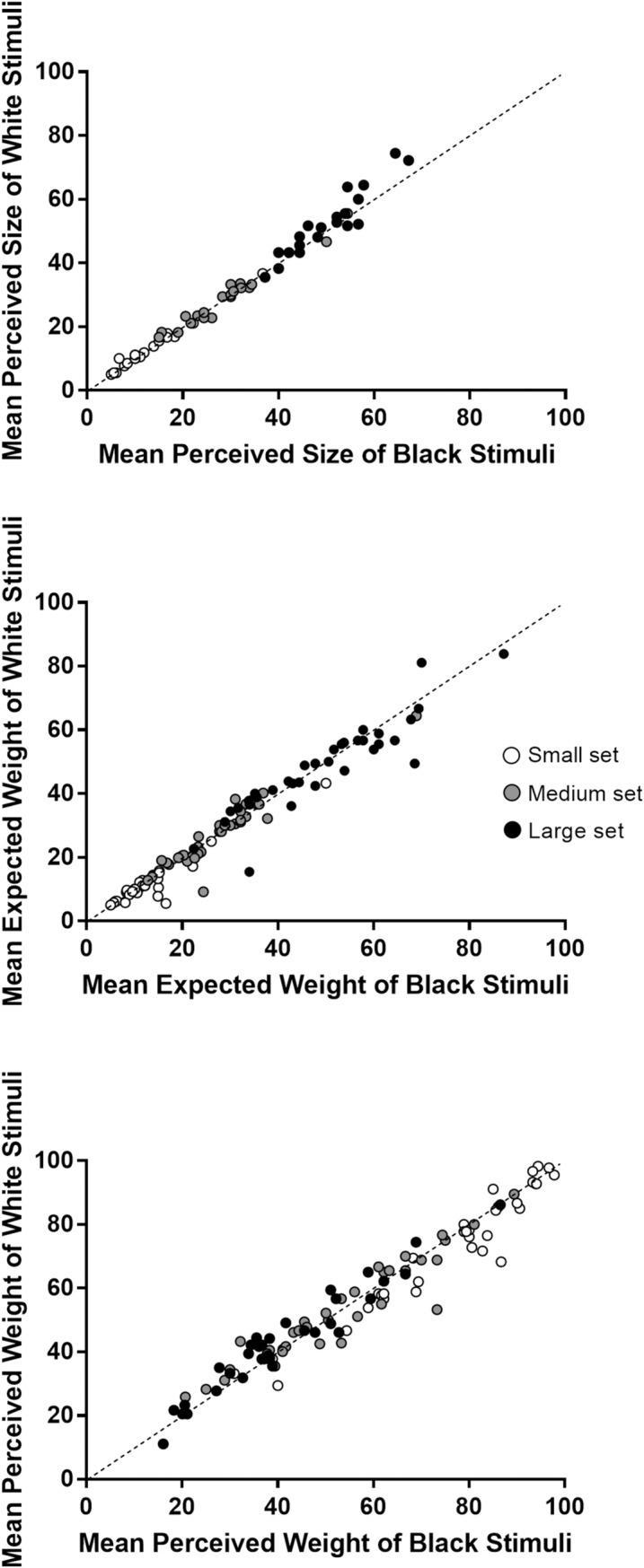
A representation of the effects of brightness on the three dependent variable at the individual level. The top, middle, and bottom panel refer to perceived size, expected weight, and perceived weight, respectively.

The main effects of brightness on perceived size, expected weight, and perceived weight were small or null. In principle, this can be due to the fact that these effects were small or null at the individual level, or to the fact that some of the participants showed a strong effect in one direction (e.g., a strong, regular brightness–weight illusion) and others showed a strong effect in the opposite direction (e.g., a strong, inverted brightness–weight illusion). In order to clarify this point, Fig 3 shows, for each participant, the mean for the white stimuli as a function of the mean of the black stimuli, relatively to perceived size (top panel), expected weight (middle panel), and perceived weight (bottom panel). The means are averaged across shape, and different colors are used to represent volume. Each point represents a participant, and a point lying below the diagonal line indicates that the mean of the dependent variable was larger for black stimuli than for white stimuli. Visual inspection of Fig 3 shows that, with a few exceptions, the points tend to cluster along the diagonal line. This suggests that, for all the three dependent variables, the effects of brightness were small and consistent across participants.

## Experiment 2

To summarize, neither the expectation model nor the hypothesis that perceived size would exert a direct influence on perceived weight can fully account for the results of Experiment 1. In Experiment 2, we sought to test the generalizability of Experiment 1’s outcomes using a different method. Specifically, we used *random conjoint measurement* (RCM), a method based on a paired comparison task in which participants have to provide a binary response [43,44,45] (for the application of a variant of the RCM paradigm in the context of weight illusions, see [25]). In order to keep the length of the experiment within reasonable limits, we used a subset of the stimuli that we had used in Experiment 1, namely the stimuli of the medium set (this choice was arbitrary).

### Participants

Twelve right-handed participants (seven females, five males) took part in the experiment. They were undergraduate students at the University of Padua, ranging in age from 21 to 34 years (*M* = 25.58, *SD* = 4.59). All had normal or corrected-to-normal vision and normal function of their upper limbs. None of them had participated in Experiment 1. They were naive as to the purposes of the experiment and received €5 for their participation. All of them participated in the perceived size, the expected weight, and the perceived weight sessions (see below).

### Stimuli and design

The stimuli were the six (3 Shape × 2 Brightness) medium-volume stimuli (i.e., 729 cc) that we used in Experiment 1. The perceived size and the expected weight sessions lasted about 15–20 minutes and took place one after the other on the same day, whereas the perceived weight session lasted about 30–35 minutes and took place at least 2 days after the first two sessions. Half of the participants first completed the perceived size session, followed by the expected weight session, and then the perceived weight session; the order of the first two sessions was inverted for the other half of the participants.

In all the three sessions, participants were presented with pairs of stimuli. Touching the stimuli was not permitted in the perceived size and expected weight sessions, whereas in the perceived weight session the participants were asked to weight the stimuli one after the other. In the perceived size session, participants were asked to indicate which of the two stimuli appeared to be larger; in the expected weight and perceived weight sessions, the participants were asked to indicate which of the two stimuli appeared to be heavier (only in the perceived weight session the participants were allowed to weigh the stimuli).

In the perceived size and expected weight sessions, the participants were randomly presented with 6 × 5 = 30 possible pairs of stimuli. The left–right order of the stimuli in each pair was counterbalanced across participants. In the perceived weight session, participants were randomly presented with two repetitions of the 30 pairs of stimuli (i.e., 60 trials in total), and for each pair, the left–right order of the stimuli was counterbalanced across repetitions. This should have prevented individual responses from being affected by a negative time order error (i.e., the phenomenon whereby, given a pair of stimuli, the perceived weight of the stimulus lifted second tends to be overestimated with respect to the perceived weight of the stimulus that is lifted first [46,47]).

### Procedure

Everything was identical to Experiment 1, except that in each trial, the participants were presented with pairs of stimuli instead of one single stimulus. For each trial, one stimulus was located on the participants’ left, and the other was on the participants’ right, at a distance of about 30 cm from each other. The stimuli were placed symmetrically with respect to the participants’ sagittal plane.

At the beginning of all three sessions, written instructions informed the participants that they would be presented with pairs of objects differing in shape and color. In the perceived size session (expected weight session), the participants were then informed that their task was to evaluate which of the two stimuli appeared to be larger (heavier). The participants were further instructed not to touch the stimuli at any moment during the experiment. In the perceived weight session, the participants were instructed to first lift the stimulus on their left and then the stimulus on their right, by grasping the string on the top of each stimulus using the thumb and index fingers of their right hand. The participants were further instructed to keep their elbows on the table during the lifting action, to avoid oscillations of the stimuli as much as possible, and to respond only after lifting the stimulus on the right. They were not allowed to repeat the lifting action. In all three sessions, the participants were instructed to respond “left” or “right” to indicate which stimulus appeared to be larger/heavier. Moreover, they were informed that, in some cases, the difference between the stimuli could be very small; therefore, they had to pay attention to subtle differences between the stimuli.

### Results and discussion

We obtained estimates of the contributions of shape and brightness to the perceived size, expected weight, and perceived weight of the stimuli by fitting an additive conjoint measurement model to the pooled data [25,44,45]. The number of stimuli and repetitions was too small to allow us to fit the model to the individual data. To define the model, we denoted the three levels of the shape factor (cube, sphere, and tetrahedron) as *s*_*C*_, *s*_*S*_, and *s*_*T*_, and the two levels of the brightness factor (white and black) as *c*_*W*_ and *c*_*B*_. Each stimulus object may be described as a pair (*s*_*i*_, *c*_*j*_), where *s*_*i*_ is a level of shape and *c*_*j*_ is a level of brightness. Consistently with the basics of the additive conjoint measurement paradigm, we presumed that the shape and brightness of a stimulus would contribute to the response variable (i.e., perceived size, expected weight, or perceived weight) according to an additive model–that is, for any stimulus *d*_*ij*_, the combined contribution would be the sum *σ*(*s*_*i*_) + *γ*(*c*_*j*_), where *σ*(*s*_*i*_) and *γ*(*c*_*j*_) are scale values associated with level *s*_*i*_ of shape and level *c*_*j*_ of brightness. Our choice of the additive response model was somewhat arbitrary because we could have equivalently used, for instance, a multiplicative response model. However, this arbitrary choice did not have any substantial implications for the study outcomes.

In each trial of the experiment, two stimuli, *d*_*ij*_ and *d*_*kl*_, were presented in a two-alternative forced choice task. Our model presumes that the response in the trial was guided by the following rule:

*d*_*ij*_ is judged lighter or heavier than *d*_*kl*_ depending on whether

*σ*(*s*_*i*_) + *γ*(*c*_*j*_) − *σ*(*s*_*k*_) + *γ*(*c*_*l*_) + *Z* is smaller or larger than 0,

where *Z* is a random noise variable with standard normal distribution. This response rule is itself consistent with the random version of additive conjoint measurement [43,44]. The additive model thus implies a set [*σ*(*s*_*C*_), *σ*(*s*_*S*_), *σ*(*s*_*T*_), *γ*(*c*_*W*_), *γ*(*c*_*B*_)] of five unknown parameters, which may take different values in different experimental conditions (perceived size vs. expected weight vs. perceived weight) and which we estimated through the maximum likelihood method. Importantly, within the frame of the RCM paradigm, the parameter estimates are determined up to linear transformations. This implies that in the additive model, one parameter for each of the two factors could be arbitrarily set to 0, reducing the number of parameters to be estimated to three. Therefore, we arbitrarily set parameters *σ*(*s*_*C*_) and *γ*(*c*_*W*_) to 0. In order to test the statistical significance of the contributions of shape and brightness to the response variables (i.e., perceived size, expected weight, or perceived weight), we compared the fit of the additive model with that of the saturated and single-factor models described below.

Besides the additive model, we tested three other models on the same experimental data. One is the saturated model–that is, a model having as many parameters as the combinations of levels in our experimental design. Therefore, this model had 3 × 2 = 6 parameters to be estimated, although this number was reduced to 5 after arbitrarily setting one of the parameters to 0. The other two models separately expressed the hypotheses that the response variable only depended on one of the stimulus factors in the experiment. Specifically, the shape-only model involved three parameters [*σ*(*s*_*C*_), *σ*(*s*_*S*_), *σ*(*s*_*T*_)], which were reduced to two by arbitrarily setting *σ*(*s*_*C*_) = 0. Symmetrically, the brightness-only model involved two parameters [*γ*(*c*_*W*_), *γ*(*c*_*B*_)], which were reduced to one by arbitrarily setting *γ*(*c*_*W*_) = 0.

#### Perceived size

The additive model proved to fit the data significantly better than the shape-only model (χ^2^(1) = 14.05, *p* < .001) and brightness-only model did (χ^2^(2) = 48.03, *p* < .001), suggesting that the contributions of both brightness and shape to perceived size were statistically significant. The saturated model did not fit the data significantly better than the additive model did (χ^2^(2) = 3.14, *p* > .10), indicating that the latter model provided an adequate fit to the data.

**Fig 4:**
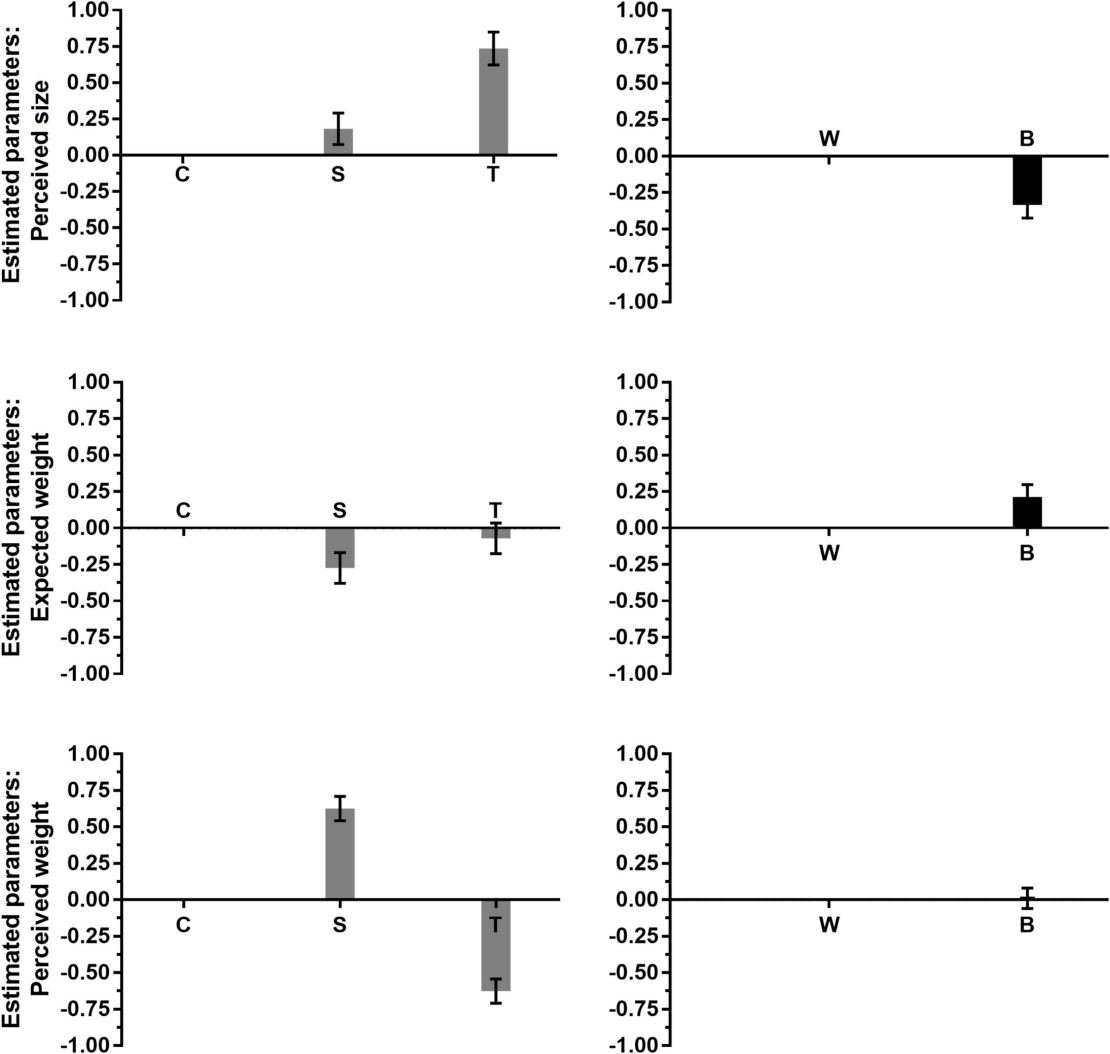
The parameter estimates for Experiment 2. The top, middle, and bottom panels refer to perceived size, expected weight, and perceived weight, respectively. The left panels represent the estimated parameters for shape; the right panels represent the estimated parameters for brightness. The letters C, S, and T stand for cube, sphere, and tetrahedron, respectively. The letters W and B stand for white and black, respectively. The error bars represent the standard error of the estimate.

The top panels in Fig 4 show the estimated parameters of the additive model (with estimated standard errors) for shape (left panel) and brightness (right panel), relatively to the perceived size session. Inspection of the parameters for shape (left panel) shows that spheres were perceived to be slightly larger than cubes, and tetrahedrons were perceived to be larger than both spheres and cubes. Considering only the 324 trials (i.e., 27 pairs × 12 participants) with pairs of stimuli differing in shape, the percentage of trials in which each shape was judged to be the largest was 22.2% for cubes, 28.8% for spheres, and 49% for tetrahedrons. Inspection of the parameters for the brightness factor (top right panel in Fig 4) shows that white stimuli tended to be judged as being larger than black stimuli. Considering only the 216 trials (i.e., 18 pairs × 12 participants) with pairs of stimuli differing in brightness, the percentage of “larger” responses was 62% for the white stimuli and 38% for the black stimuli.

The results are remarkably similar to those of the perceived size session in Experiment 1, since they show that tetrahedrons were perceived to be larger than the other two shapes, that spheres were perceived to be slightly larger than cubes, and that white stimuli were perceived to be larger than black stimuli. These findings support the generalizability of the effects of shape and brightness on perceived size, as these effects appear to be consistent across different samples of participants, different sets of stimuli, and different psychophysical methods.

#### Expected weight

The additive model fit the data significantly better than the shape-only model (χ^2^(1) = 6.11, *p* = .013) and better than the brightness-only model (χ^2^(2) = 7.31, *p* = .026), suggesting that the contributions of both brightness and shape to expected weight were statistically significant. The saturated model did not fit the data significantly better than the additive model did (χ^2^(2) = 1.44, *p* > .10), indicating that the latter model provided an adequate fit to the data.

The middle panels in Fig 4 show the estimated parameters of the additive model (with estimated standard errors) for shape (left panel) and brightness (right panel), relatively to the expected weight session. Inspection of the parameters for shape (left panels) shows that cubes and tetrahedrons had approximately equal expected weights, and that both were expected to be heavier than spheres. Considering only the 324 trials with pairs of stimuli differing in shape, the percentage of trials in which each shape was judged to be the heaviest was 37.8% for cubes, 27.1% for spheres, and 35.1% for tetrahedrons. Inspection of the parameters for the brightness factor (middle-right panel in Fig 4) shows that black stimuli were expected to be heavier than white stimuli. Considering only the 216 trials with pairs of stimuli differing in brightness, the percentage of “heavier” responses was 41.7% for the white stimuli and 58.3% for the black stimuli.

Recall that in Experiment 1, judgments of expected weight were mediated by perceived size, as tetrahedrons were perceived to be larger and expected to be heavier than both spheres and cubes, and spheres were perceived to be larger and expected to be heavier than cubes. In the expected weight session of Experiment 2, we obtained a clearly different pattern of results with respect to the expected weight session of Experiment 1, notwithstanding that the judgments of perceived size were remarkably similar in the two experiments. A plausible explanation for this finding is that variations in perceived size were particularly salient in Experiment 1, since the stimuli varied not only in shape and brightness but also in volume. This might have prompted the participants to base their judgments of expected weight on perceived size. In line with this reasoning, by keeping volume constant, we decreased the salience of variations in perceived size in Experiment 2, thus obtaining a dissociation between expected weight and perceived size. This hypothesis is further supported by the fact that, differently from Experiment 1, we obtained a clear negative relationship between brightness and expected weight in Experiment 2, since black stimuli were expected to be heavier than white stimuli. This is inconsistent with the results of Experiment 1, in which the main effect of brightness on expected weight failed to emerge, probably because the negative relationship between brightness and expected weight was overridden by the positive relationship between brightness and perceived size. The mismatch between the results of Experiments 1 and 2 provides further support to the hypothesis that judgments of expected weight were not mediated by perceived size in Experiment 2.

#### Perceived weight

The model that best described participants’ responses in the perceived weight session was the shape-only model. Indeed, the additive model fit the data significantly better than the brightness-only model (χ^2^(2) = 225.2, *p* < .001), but it did not fit the data significantly better than the shape-only model (χ^2^(1) = 0.03, *p* > .10). Moreover, the saturated model did not fit the data significantly better than the shape-only model (χ^2^(3) = 0.65, *p* > .10). In sum, the results indicate that shape had a statistically significant effect on perceived weight, whereas brightness did not.

The bottom-left panel in Fig 4 shows the estimated parameters of the shape-only model (with estimated standard errors). For the sake of comparison, we also represented the estimated parameters of the additive model for brightness in the bottom-right panel, although it is important to remark that the shape-only model fit the data significantly better than the additive model. Inspection of the parameters for shape (left panel) shows that spheres were perceived to be heavier than cubes and tetrahedrons, and cubes were perceived to be heavier than tetrahedrons. Considering only the 648 trials (i.e., 54 pairs × 12 participants) with pairs of stimuli differing in shape, the percentage of trials in which each shape was judged to be the heaviest was 33.3% for cubes, 54.3% for spheres, and 12.3% for tetrahedrons. Moreover, considering only the 432 trials (i.e., 36 pairs × 12 participants) with pairs of stimuli differing in brightness, the percentage of “heavier” responses was 49.8% for the white stimuli and 50.2% for the black stimuli. The latter result indicates the absence of an effect of brightness on perceived weight.

In sum, the results of the perceived weight session were similar to those of the perceived weight session of Experiment 1. In both experiments, spheres were perceived to be heavier than the other two shapes, and cubes were perceived to be heavier than tetrahedrons. Additionally, brightness did not appear to have any clear effects on perceived weight in either experiment. Since the two experiments were conducted with different samples of participants, different sets of stimuli, and different psychophysical methods, the similarity between the patterns of results supports the robustness and generalizability of the visual shape–weight illusion. The comparison between expected and perceived weight reveals some noteworthy discrepancies from the predictions of the expectation model, although these discrepancies were different from those in Experiment 1. Specifically, in Experiment 2, cubes were both expected and perceived to be heavier than tetrahedrons, and black stimuli were not perceived to weigh less than white stimuli, despite the former clearly expected to be heavier than the latter. The results are also partially at odds with the hypothesis of a direct influence of perceived size on perceived weight, since spheres were perceived to be both larger and heavier than cubes, and white stimuli were not perceived as weighing less than black stimuli, despite white stimuli being perceived as larger than black stimuli.

## General discussion

A robust and reliable visual shape–weight illusion emerged in both a numerical rating task (Experiment 1) and a paired comparison task (Experiment 2). Specifically, when the data were averaged across factors volume and brightness, we found that spheres were perceived to be heavier than cubes and tetrahedrons, and cubes were perceived to be heavier than tetrahedrons. This pattern of results is similar to that obtained by Kahrimanovic, Bergmann Tiest, and Kappers [27,28] in studies on the haptic shape–weight illusion. Although further studies are required to directly compare of the magnitude of the haptic and the visual shape–weight illusion, the results suggest that the shape–weight illusion can be elicited using only visually-derived shape information. In contrast, we found no reliable evidence of a brightness–weight illusion in Experiment 1 or 2. Specifically, in Experiment 1, we found a regular brightness–weight illusion for the large stimuli, an inverted brightness–weight illusion for the small stimuli, and no brightness–weight illusion for the medium-sized stimuli. The lack of a brightness–weight illusion for the medium-sized stimuli was replicated in Experiment 2, in which a paired comparisons method was used.

As regards the brightness–weight illusion, our results are inconsistent with those reported by Walker, Francis, and Walker [30], who showed, in their Experiment 1, that a white sphere had a probability of about 0.8 to be judged as heavier than a black sphere of identical size and weight. Even though we used a method similar to that employed by Walker, Francis, and Walker [30] in Experiment 2, we found that white stimuli had a probability of only 0.49 to be judged as heavier than black stimuli. The discrepancy between the results of the two studies might be due to differences in the physical weight of the stimuli (i.e., 220 g in our study versus 129 g in Walker, Francis, and Walker [30]) and the mode of lifting (i.e., by means of a string in our study versus by holding the stimulus in the hand in Walker, Francis, and Walker [30]). Concerning the first point, according to Weber’s law, participants’ sensitivity to differences in weight tends to be higher for relatively light stimuli than for relatively heavy stimuli. Therefore, in Walker, Francis, and Walker’s [30] study, the use of relatively light stimuli might have enhanced the participants’ sensitivity to subtle differences in weight. Concerning the second point, the results obtained by Ellis and Lederman [21] suggest that participants are more sensitive to subtle weight differences when they are asked to lift the stimuli by grasping them (like in Walker, Francis, and Walker [30]) than when the stimuli are lifted by means of a string or a pulley (as in our study). Independently of the exact origins of the discrepancy between our results and those obtained by Walker, Francis, and Walker [30], it is important to note that the current study suggests that the existence of the brightness–weight illusion is restricted to a specific weight range and a specific mode of lifting. Therefore, the brightness–weight illusion is less robust and generalizable than the size–weight, material–weight, and shape–weight illusions (see also [29,32]).

### Explicit and implicit weight expectations

The results of Experiments 1 and 2 are only partially consistent with the predictions from the expectation model. Consistent with those predictions, tetrahedrons were expected to be heavier and perceived to be lighter than spheres in both experiments. Moreover, in Experiment 1, tetrahedrons were expected to be heavier and perceived to be lighter than cubes. Inconsistent with the expectation model’s predictions, spheres were both expected and perceived to be heavier than cubes in Experiment 1, and tetrahedrons were perceived to be lighter than cubes in Experiment 2, despite tetrahedrons and cubes having approximately equal expected weight. As regards to the influence of brightness on perceived weight, inconsistent with the expectation model’s predictions, in Experiment 1, we found that the small black stimuli were both expected and perceived to be heavier than the small white stimuli and that the large black stimuli were perceived to be lighter than the large white stimuli, despite the black and white large stimuli being approximately equal in expected weight. Also, inconsistently with the expectation model’s predictions, we found that black and white stimuli were approximately equal to each other in perceived weight in Experiment 2, despite black stimuli being expected to be heavier than white stimuli.

Judgments of expected weight were clearly inconsistent across the two experiments. Specifically, in Experiment 1, tetrahedrons were expected to be heavier than spheres and cubes, and spheres were expected to be heavier than cubes. By contrast, in Experiment 2, tetrahedrons and cubes were expected to be approximately equal to each other in expected weight, and both were expected to be heavier than spheres. The effects of brightness on expected weight were also inconsistent across the two experiments, as Experiment 1 showed no clear relationship between brightness and expected weight for the medium-volume stimuli, whereas such a relationship clearly emerged in Experiment 2. A plausible explanation for the mismatch between the expected weight judgments in the two experiments is that in Experiment 1, such judgments were prominently driven by perceived size, whereas this was not the case in Experiment 2. This could be because in Experiment 1, variations in perceived size were made particularly salient by the manipulation of the stimuli’s volume. If this hypothesis is correct, then judgments of expected weight would be driven not only by the stimuli’s properties but also by flexible task-dependent strategies that depend on the experimental design’s features, such as the saliency of a specific stimulus property. In this regard, it is perhaps worth emphasizing that the method that we used to measure expected weight has been widely used in previous studies [22,23,25,29,30]; nevertheless, the visual estimation of the weight of artificial and unfamiliar objects like those we used in the current study can be a hard task for the participants, which can possibly explain why their responses were driven by flexible heuristic strategies. It is also worth noting that, differently from judgments of expected weight, judgments of perceived size and perceived weight were remarkably consistent across the two experiments. This suggests that perceived size and perceived weight are relatively stable psychological properties that are more related to the properties of the stimuli than to the structure of the experimental design. In other words, perceived size and perceived weight might be the result of relatively stable perceptual processes, rather that the result of flexible heuristic strategies like explicit reports of expected weight.

The observed effects of shape on perceived weight are at least partially inconsistent with the predictions from the expectation model. Buckingham and colleagues [12,17,22] recently proposed a revision of the expectation model, according to which weight perception is affected by implicit, rather than explicit, weight expectations. Explicit weight expectations can be directly measured by asking participants to predict the weight of an object based on its visual appearance, as we did in the present study. Implicit weight expectations would instead be impervious to consciousness and would depend on ontogenetic and phylogenetic development. Consistently with this hypothesis, it can be argued that the shape-weight illusion depends on implicit rather than explicit weight expectations. Therefore, independently of explicit weight expectations, tetrahedrons would be implicitly expected to be heavier than both cubes and spheres, and cubes would be implicitly expected to be heavier than spheres. Moreover, brightness would not have any substantial influence on implicit weight expectations, even when it has a clear effect on explicit weight expectations. This hypothesis may potentially explain the mismatch between our results and the predictions from the expectation model. Nevertheless, further studies appear necessary to clarify the origins of the mismatch between explicit and implicit weight expectations, and to provide a reliable measure of implicit weight expectations. On passing, we note that a distinction between explicit and implicit expectations has also been proposed in other fields of research; for instance, it has been suggested that people have accurate implicit expectations, but inaccurate explicit expectations, about gravitational motion [48,49].

### A dissociation between the processing of cues to size?

Saccone and Chouinard [19] recently argued that one reason why perceived weight may not match the predictions from the expectation model is because the size of the stimuli is processed directly and automatically by the magnocellular pathway. Therefore, size would strongly affect perceived weight, even when it exerts a relatively small influence on reported, explicit expected weight. The results of Experiments 1 and 2 are not fully consistent with the hypothesis that perceived weight resulted from the contrast between actual weight and perceived size. Indeed, in both experiments, spheres tended to be perceived as heavier than cubes, despite spheres being perceived as larger than cubes, and no differences in perceived weight emerged between black and white stimuli, despite white stimuli being perceived as larger than black stimuli. Specifically, the results of Experiments 1 and 2 appear to be consistent with the hypothesis that perceived weight depended on the contrast between actual weight and surface area. Indeed, for a fixed physical volume, the surface area of a tetrahedron is larger than that of cube and of a sphere, and the surface area of a cube is larger than that of a sphere. This might explain why tetrahedrons were perceived to be lighter than cubes and spheres and why cubes were perceived to be lighter than spheres. Surface area also affected the perceived size of the stimuli (see also [31]), but surface area alone cannot explain why white stimuli were perceived to be larger than black stimuli or why spheres were perceived to be larger than cubes (for a given level of physical volume, the surface area of spheres was smaller than that of cubes). As regards the latter results, it is worth noting that for each level of physical volume, the vertical height of the sphere was larger than that of the cube (see Table 1). Therefore, the fact that spheres were judged to be larger than cubes is consistent with the well-known elongation bias, which refers to the phenomenon whereby when two objects of the same physical size are compared in terms of visually perceived size, the higher object usually tends to be judged as larger than the other object [50,51,52].

In sum, it can be hypothesized that a dissociation exists between the processing of the size cues that contribute to perceived weight and that of the size cues that contribute to the conscious experience of perceived size. Although this hypothesis remains rather speculative at the current stage of research on weight illusions, it appears to be worthy of further exploration because it puts into question the widely shared idea that the size–weight illusion results from the contrast between actual weight and perceived size. This hypothesis also highlights the importance of studying the relationship between size cues that contribute to perceived size and those that contribute to perceived weight (e.g., the relationship between volume and surface area in determining perceived size and perceived weight).
